# Process mining with real world financial loan applications: Improving inference on incomplete event logs

**DOI:** 10.1371/journal.pone.0207806

**Published:** 2018-12-31

**Authors:** Catarina Moreira, Emmanuel Haven, Sandro Sozzo, Andreas Wichert

**Affiliations:** 1 School of Business and Research Centre IQSCS, University of Leicester, Leicester, United Kingdom; 2 Faculty of Business Administration, Memorial University, Newfoundland and Labrador, Canada; 3 Instituto Superior Técnico and INESC-ID, University of Lisbon, Lisbon, Portugal; Central South University, CHINA

## Abstract

In this work, we analyse and model a real life financial loan application belonging to a sample bank in the Netherlands. The event log is robust in terms of data, containing a total of 262 200 event logs, belonging to 13 087 different credit applications. The goal is to work out a decision model, which represents the underlying tasks that make up the loan application service. To this end we study the impact of incomplete event logs (for instance workers forget to register their tasks). The absence of data is translated into a drastic decrease of precision and compromises the decision models, leading to biased and unrepresentative results. We use non-classical probability to show we can better reduce the error percentage of inferences as opposed to classical probability.

## 1 Introduction

In recent years, we have witnessed a vast increase in information. Given that the price of storage devices has been decreasing throughout the years, storing millions of records of information has become a common and affordable task. These large amounts of data pose serious difficulties in the extraction of valuable information, and the analysis of these datasets has become an extremely complex task. Companies often do not have control of the underlying processes that make up their products or services. This translates in workflow sequences with several redundant tasks, which play a crucial role in increasing the amount of expenses a company incurs and delays the delivery of a final product or service to a client.

In this paper, we have as objective to model a real life financial event log of a loan application belonging to a sample bank in the Netherlands. The event log is robust in terms of data, containing a total of 262,200 event logs, belonging to 13,087 credit applications. The only information known is that a customer selects a certain amount of money and submits her / his request to the bank’s web platform. Some automatic tasks are triggered and one can verify if an application is eligible for credit. The underlying tasks of this loan application are heterogeneous and consist of a mixture of computer generated processes and manual human tasks. The identification of the underlying processes that lead to a product / service is a very important task and an active research field in the scientific community, more specifically in the domain of *Business Process Management* (see, e.g., [1]).

This work is motivated by the Business Process Intelligence (BPI) challenge of 2012 (please see http://www.win.tue.nl/bpi/doku.php?id=2012:challenge). In this challenge, institutions anonymously provide real-world event logs and participants are asked to analyse the data using any techniques available that they think are suitable for the task. A jury then evaluates the best report submitted and the winning participant receives a prize. In this challenge, the owner of the financial institution data was interested in all valuable information that could be extracted from the dataset as well as several different specific aspects. Some of these aspects were concerned with the understanding of a general process that could represent the data and how decisions could influence and have impact in this process. In this work, we try to address some of these issues by exploring an alternative probabilistic graphical model (Bayesian Networks), that enables a graphical and unique analysis of how the information of some decisions taken could propagate and influence whether a client gets a credit approved or not.

### 1.1 Business process management

Defined as the set of techniques responsible for the optimization of a company’s business processes, *Business Process Management* promises the automatic detection of redundant tasks, cycles or unprofitable sequences of events, leading to an increase in the company’s productivity, efficiency and a reduction of operational costs. Under these circumstances, a business process is understood as a collection of tasks that are linked and executed in a sequence until they result in a product or a service delivered to a client (see [2, 3]).

One of the techniques used in Business Process Management (and which will be the focus of this work) is *Process Mining*. Process mining is a technique that enables the automatic analysis of business processes based on event logs. Instead of designing a workflow, process mining consists in gathering the information of the tasks that take place during the workflow process and storing that data in structured formats called *event logs* [4]. While gathering this information, it is assumed that (1) each event refers to a task in the business process; (2) each event is associated with an instance of the workflow and; (3) since the events are stored by their execution time, it is assumed that they are sorted [5]. This means that the ordering of the activities can be described by causal relationships, suggesting that decision models capable of representing cause/effect relationships are suitable models for the representation and analysis of the company’s workflow and business process. Probabilistic graphical models, such as Bayesian Networks, are examples of decision models which are capable of representing influences or causal relationships between events [6].

### 1.2 The problem of missing data

Event logs are the main source of data for the discovery of the business processes that make up a company. However, it is quite common that event logs are incomplete with several amounts of missing information (for instance, workers forget to register their tasks, system crashes, etc.). Usually, statistical methods are applied to the existing data, in order to create knowledge and overcome the missing data. However, most of the statistical methods require a complete dataset (or at least a dataset sufficiently robust) in order to perform accurate predictions [7]. The absence of data is translated into a drastic decrease of precision and compromises the statistical model, leading to biased and unrepresentative results. This affects all fields of knowledge ranging from genetics [8], psychology [9], medical research [10], etc.

Missing data involves (or leads to) high levels of uncertainty. Although many tasks are automated in corporations, there is also a significant human component in these tasks. When workers need to make decisions under scenarios with high levels of uncertainty (when data is missing, untrusted information, or simply decisions under pressure), the work force is subject to human judgment errors and such errors can lead to redundant tasks in companies or lead to more unnecessary and more complex sequences of tasks. All of this can cause additional operational costs to companies whilst also adding to the potential of increased inaccurate decisions (see [11]). The theme of human judgment errors, is covered by a large body of work which reports ample experimental evidence demonstrating that humans constantly violate the laws of classical probability theory and logic in decision scenarios under uncertainty. All of this has led to a set of well publicised decision paradoxes and fallacies (see [12–17]).

### 1.3 Non-classical probability

Classical probability theory (also called Kolmogorovian probability [18]) can sometimes have difficulty in providing effective models that can capture human judgments and decisions. Well known paradoxes like the Ellsberg paradox [19], attest to this. In order to accommodate decision paradoxes, a new discipline has emerged in the last decade, often known under the generic name of quantum cognition. This new field aims to build cognitive models by using the mathematical principles of quantum mechanics, and by so doing it uses non-classical probability (see, e.g., [20–25]). From the outset, two caveats need pointing out:

1. it is important to stress that this new approach is essentially limited to the borrowing of a formalism from quantum mechanics. Current research in this new area of work does overall, not pretend to claim that human decision-making is quantum mechanical in nature. We will therefore in the sequel of this paper often use the term ‘quantum-like’.
2. the quantum probabilistic formalism from basic quantum mechanics is by no means the only expression of non-classical probability. There exist several deviations from classical probability (those are often termed as ‘non-Kolmogorovian’ probabilistic frameworks)

In a classical setting, probability is computed using the law of total probability. Let *A* be a random variable defined by real numbers and contained in a sample space *ω*, and let *B*_*i*_ with *i* = 1, … *N* be a partition of the same sample space, then the classical law of total probability is

$$\begin{array}{c} Pr\left(A\right)=\sum_{i=1}^{N}Pr\left(B_{i}\right)Pr\left(A|B_{i}\right) \end{array}$$ (1)

Quantum cognition does not use classical probability theory. In quantum cognition, probabilities are defined by complex numbers, instead of real numbers, and they are called amplitudes (we denote them by *ψ*). A complex number is a number that can be expressed in the form *z* = *a* + *ib*, where *a* and *b* are real numbers and *i* corresponds to the imaginary part, such that *i*^2^ = −1. A complex number can also be described in the form *z* = |*r*|*e*^*iθ*^, where $\left|r\right|=\sqrt{a^{2}+b^{2}}$. The *e*^*iθ*^ term is defined as the phase of the amplitude. These amplitudes are related to classical probability by taking the squared magnitude of these amplitudes through the so called Born rule [26]. This is achieved by multiplying the amplitude with its complex conjugate (Eq 2)

$$\begin{array}{c} Pr\left(A\right)={\left|\sum_{i=1}^{N}\psi \left(B_{i}\right)\psi \left(A|B_{i}\right)\right|}^{2} \end{array}$$ (2)

From a strictly physics point of view, a consequence of using Born’s rule to define probabilities will lead to the emergence of quantum interference effects. If we expand Eq 2, we will end up with a quantum probability formula (i.e. a non-classical probability formula), which contains two terms: one that corresponds to the classical probability and another term that corresponds to the quantum interference effects (see, e.g., [27])

$$\begin{array}{c} Pr\left(A\right)=\sum_{i=1}^{N}Pr\left(B_{i}\right)Pr\left(A|B_{i}\right)+interference \end{array}$$ (3)

By manipulating the quantum interference term, we can disturb the classical probability values through constructive interferences (when the interference term is positive) or destructive interferences (when the interference term is negative).

In terms of constructing *inferences* out of the missing data problem in business process management, we can claim that quantum probabilistic inferences can be considered as an additional layer to classical probability inferences allowing for a non-linear parameterisation of the data. Our hypothesis is thus that one can take advantage of this additional parametric layer and use it to improve the results of decision models in business process management. This then will lead to more robust decision scenarios that can help reduce operational costs in companies by reducing insignificant tasks and consequently improve the service delivery times to clients.

To date, the literature has shown that quantum cognitive models are able to accommodate many paradoxical situations in a general and fairly straightforward framework (see, e.g., [24, 28–32]). There are also quantum predictive models that are able to predict the outcome of these decision scenarios with low percentage errors (see, e.g., [33, 34]). However, current quantum cognitive models have been applied in very simple decision scenarios (for instance, the Prisoner’s Dilemma), which can be modelled with at most two random variables. To the best of our knowledge, no quantum-like model has ever been applied in the context of a complex real life decision scenario, such as in Business Process Management.

### 1.4 Why Bayesian networks?

In process mining, there are many models used in the literature, which range from Markov Chains [35, 36], to Petri Nets [37], Neural Networks [38] and even to BPMN [39]. Markov Chains and Petri Nets are probably the most used models in the literature of process mining [40], since they can make an easy and direct mapping from the event logs to a causal and sequential structure (for more information on how these models can be applied, please see [41]). Bayesian Networks, on the other hand, differ from Markov Chains, because of their cycle-free and directed structure. They have the advantage of dealing with uncertainty differently from Markov Chains. While in Markov Chains business processes are modelled as a chain of events that are observed to occur, under a Bayesian Network perspective, this does not apply: each task can either be present or absent in the business process. Therefore, Bayesian Networks allows the modelling of uncertainty associated with a business process by performing a different analysis that will enable the computation of the probability of some task of the business process occurring, given that we do not know which tasks have already been performed [42]. It is this capability and graphical analysis of dealing with uncertainty that make Bayesian Networks attractive models in many research fields including medical decision-making [43] and risk management [44]. As shown throughout the paper, the graphical analysis that a Bayesian Network can perform is something that is not directly perceived in a Markov Chain and this is indeed one contribution of the present paper. Bayesian Networks could also be used to assist business managers in decision-making by providing them a visual and probabilistic analysis of a decision-scenario.

We are aware that the directed acyclic structure of the Bayesian Network leads to a simplification of the business process itself. But since the goal of this paper is to show a different and alternative probabilistic model to compute inferences under high levels of uncertainty, this does not hold a significant drawback. The analysis that we performed compares inferences in the classical Bayesian Network with a non-classical Bayesian Network with an incomplete dataset. For the validation of the probabilistic inferences, we used a classical Bayesian Network with the full dataset as ground truth.

### 1.5 What are the main contributions of this paper?

The applicability of quantum-like models in complex real life scenarios, such as in medical decision-making problems or decision-making in economical / financial scenarios, is still an open research question in the literature. As of yet, to the best of our knowledge, no such studies have been conducted. For this reason, the purpose of this paper is to provide for a first step into this direction. We want to test the effectiveness of quantum-like cognitive models in a real life financial scenario corresponding to a Dutch bank, which provides credit loans to its clients. We focus our attention on the issue of event logs which are incomplete and which thus can lack a large amount of data. The main contribution of our paper is the study of the impacts of missing data in the reconstruction of the institution’s business processes. We investigate how classical probabilistic models are affected by missing data and we explore a non-classical probability approach. We focus on the use of quantum-like probabilistic inferences as an alternative mathematical model to the classical probability model.

In a nutshell, the paper aims to contribute to:

1. **optimizing** the institution’s business processes by identifying and eliminating redundant tasks. This leads to an exponential drop in the *costs* and *time* that are involved in the loan application.
2. **the extraction of a decision model** which is representative of an optimised loan application.
3. **dealing with missing data** by exploring the impact of two different probabilistic inference frameworks (one based on classical probability theory and the other based on non-classical (here quantum theory based) probability).

### 1.6 Organization of the paper

Given the complexity of the problem at hand, our paper is segmented and organized in the following topics:

- Processing of the event log by discovering the underlying information that makes up the event log and making sense of the relevance of this information for the construction of the business process (Section 2.1).
- Extraction of the institution’s business process by extracting the sequence of tasks involved in each loan application from the bank’s event log and by detecting redundant and misconducted tasks (Section 2.2);
- Construction of a decision model representative of the business process extracted. There are many options to be explored here. In this paper, we opt for Bayesian Networks (Section 4);
- Investigation of the impact of missing data in the event log for classical and non-classical probabilistic inferences. We explore alternative mathematical approaches to deal with uncertainty that are not based in classical probability theory. Again, there are many non-Kolmogorovian probabilistic frameworks. Due to the recent successful application of quantum-like models (see, e.g., [20]), we will investigate quantum-like probabilistic inferences (Section 4.2).

## 2 Case study: A loan application bank in the Netherlands

The event log that we use in this work is taken from a bank in the Netherlands and corresponds to a loan application, where customers request a certain amount of money. This dataset has been provided for the BPI Challenge in 2012 and is publicly available (BPI Challenge 2012 Dutch financial institution Dataset: http://www.win.tue.nl/bpi/doku.php?id=2012:challenge). The loan application starts with a webpage from where a customer selects a certain amount of money and then submits his request. Then, the application performs some automatic tasks and checks if an application is eligible. If it is eligible, then the customer is sent an offer by mail (or by phone). After this offer is received, it will be evaluated. In case of any missing information, the offer goes back to the client and is again evaluated until all the required information is gathered. A final evaluation is then performed and the application is then approved [45].

It is also known that the process is composed of three different groups of processes. The first letter of each task corresponds to an identifier of the sub-process it belongs to. The tasks that start with the letter *A* correspond to states of the application, which are computer automated tasks. The tasks that start with the letter *O* correspond to offers, which are communicated to the client. It is not clear from the dataset if these tasks are automatically generated by the application or if they involve any human work. The tasks that start with the letter *W* correspond to the work item belonging to the application and correspond to human tasks.

### 2.1 Processing the event log

The event log consists of a structured file, which requires a substantial amount of processing effort in order to identify and extract all relevant information for the analysis. In total, we identified 262,200 events, which are contained in 13,087 different loan applications. Each loan application is associated with some amount of money requested by the client. The summary of all the different tasks extracted from the event log are laid out throughout Tables 1 to 3.

**Table 1:** System application tasks that were identified during the processing of the event log. Some redundant task were identified, but still not confirmed: {A_SUBMITTED, A_PARTLYSUBMITTED} and {A_APPROVED, A_REGISTERED, A_ACTIVATED} [45].

Event	Occurrences	Description
**A_SUBMITTED** **A_PARTLYSUBMITTED**	13 087 13 087	Initial states. All 13 087 cases recorded in the log file start with these events. These tasks correspond to the action of a client starting the submission for a request of some amount of money to be loaned.
**A_PREACCEPTED**	7 367	The application has not been accepted, because it requires additional information.
**A_ACCEPTED**	5 113	The application has been accepted and ready to go to the final stage. However, it can still need some additional information from the client.
**A_FNIALIZED**	5 015	The submitted application is fully accepted and ready for assessment.
**A_CANCELLED** **A_DECLINED**	2 807 7 635	End states of an unsuccessful application process. Not clear what is the difference between them.
**A_APPROVED** **A_REGISTERED** **A_ACTIVATED**	2 246 2 246 2 246	Represent the end of a successful application process. These three events always appear together interchangeably and correspond to an approved loan application.

**Table 2:** Worker tasks that were identified during the processing of the event log. Workers tasks mean that these tasks are pure manual and performed by humans [45].

Event	Occurrences	Description
**W_Calling after sent offers**	52 016	Event triggered whenever there is an offer sent to a client
**W_Assessing the application**	20 809	Evaluates whether the application is elicit for credit
**W_Filling in information**	54 850	Required after applications are pre accepted
**W_Fixing incoming lead**	16 566	Triggered by the initial application processes and whenever a client did not fill all the required information
**W_Calling to add missing information**	25 190	Additional information needed after performing the application assessment
**W_Rate fraud**	664	Triggered after the assessment of the application, it is investigated cases of suspicious fraud
**W_Change contract details**	0	Triggered when it is required a change in the contract

**Table 3:** Tasks corresponding to offers that were identified during the processing of the event log. These tasks are not fully known if they are conducted by works, by automatic application processes or by a mix of both [45].

Event	Occurrences	Description
**O_CREATED** **O_SELECTED** **O_SENT**	7 030 7 030 7 030	Offer created for the client The client was selected to receive an offer Offer sent to the client
**O_SENT BACK**	3 454	Client’s response to the received offer
**O_ACCEPTED**	2 243	Corresponds to an end state of a successful offer Both parties agree with the offer.
**O_CANCELLED** **O_DECLINED**	3 655 802	Corresponds to end states of an unsuccessful offer. Either the client or the institution rejected the offers or the offer was cancelled for some reasons

Table 1 summarises the computed automated tasks, *A*_. These tasks correspond to the bank application, and from what the data informs us of, the costumer triggers the initiation of the process by submitting some required amount of money. The root node of the entire application was identified as being *A*_*SUBMITTED*. At this stage, we already identified some redundancy in the data since it seems that the processes *A*_*SUBMITTED* *and* *A*_*PARTLYSUBMITTED* always occur together and in sequence. This means that the bank application has an additional process that is unnecessarily consuming time and computer resources. However, we can only confirm this redundancy after analysing the graphical structure of the process (Section 2.2). The same redundancy was found at the end of the application process. The three redundant end nodes identified were *A*_*APPROVED*, *A*_*REGISTERED* and *A*_*ACTIVATED*. These three events always occur together interchangeably.

Table 2 summarises the tasks that correspond to manual workers. The event log contains a time sequence information regarding these tasks, which can either be *START*, *SCHEDULE* or *COMPLETE*. As the name indicates, *START* corresponds to the beginning of a worker’s task. When the worker has finished addressing the task, then the event state is changed to *COMPLETE*. Tasks that are postponed to some specified date (or time) are marked *SCHEDULE*. For the analysis of the event log and for the extraction of the business process, we only considered the tasks that were in the state *COMPLETE*. Since these tasks are purely performed by humans, a lot of errors are expected. For instance, the task *W*_*Change contract details* exists on the system, however it has never been performed by any worker in the bank.

Table 3 summarises the tasks that correspond to Offers. It is not clear from the dataset or from the information provided if these tasks correspond to human tasks or to application tasks. We are guessing that they are a mix of both, but we will never know this with certainty. For what we understood from the process, whenever a loan application is elicited for credit, an offer is created and sent to the client. This offer can be sent back to the client, presumably if some changes are needed to the offer. It can also be accepted if the client accepts the offer or it can be cancelled or declined. Regarding these last two eventualities, it is not clear what the difference is between them. It is supposed that an offer can be declined if the client or the institution rejects the offer. Three possibly redundant tasks were also identified, given that they always appear together: *O*_*CREATED*, *O*_*SELECTED* and *O*_*SENT*. Again, this redundancy of tasks can contribute to a drop in the productivity of the service by consuming extra resources and time.

In summarizing, the dataset contained a total of 262,200 events, which are contained in 13,087 different loan applications. We identified 24 different events and several redundant events that could be subject to some degree of optimization.

### 2.2 Extracting a business model

**Fig 1:**
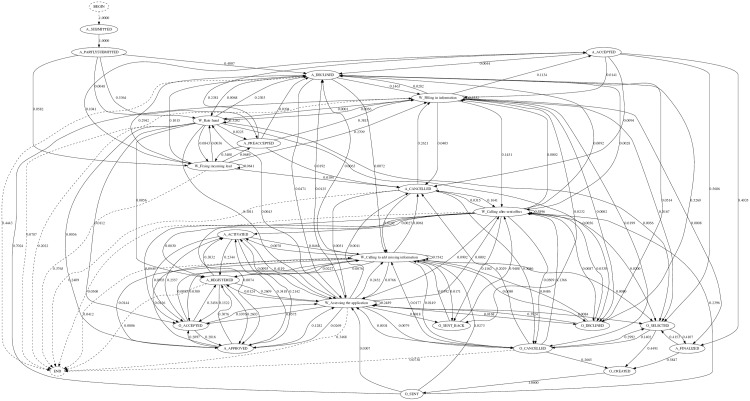
Extracted business process from a Dutch’s financial institution Dataset.

In a first step towards the understanding of the company’s business processes, we generated a graphical model showing the sequence of all tasks that were conducted from the beginning of the loan application request, until its end (either with a successful application or with a rejection). The resulting plot shows a graphical structure where each node represents a task and each edge represents the probability of transiting from one task to another (Fig 1). Fig 1 shows the complexity of the business process extracted directly from the event log. One can see that the extracted graph is incomprehensible, very dense and extremely unclear. For this reason, we needed to take some steps in order to extract information and value out of this process.

**Fig 2:**
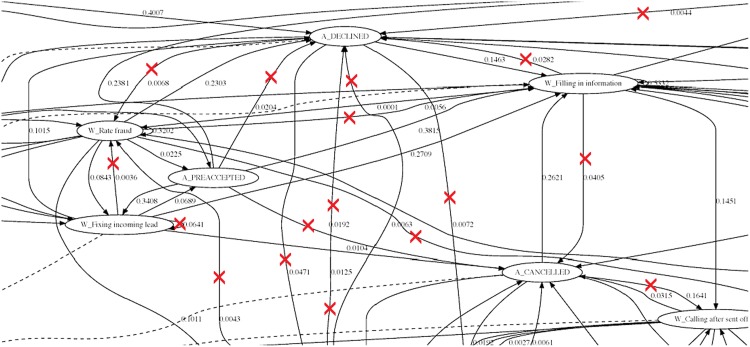
Part of the business process extracted where we identify and remove very rare sequences of tasks. We consider that a sequence is rare if the probability of its occurrent is bellow 0.05.

To extract informational value out of the business process, we removed a sequence of tasks that were very unlikely to occur. In other words, tasks that had very small transition probabilities. Consider Fig 2, which is a representation of a subset of the business process in Fig 1. For instance, the probability of executing the sequence of tasks *A*_*DECLINED* → *W*_*RateFraud* is 0.0068. Since the occurrence of the sequence of these tasks is very rare, one can ignore it and discard it from the analysis. In this paper, any sequences of tasks with a transition probability below 0.05 are not deemed relevant to assess the value of the internal processes conducted in the company. Consequently, those sequences were ignored.

### 2.3 Elimination of redundant tasks

**Fig 3:**
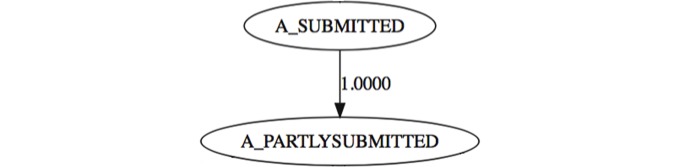
Redundancy found between events {A_SUBMITTED, A_PARTLYSUBMITTED}.

When identifying the business processes from the event log, we suspected that there were several tasks, which were redundant and could be merged into a single task. Regarding the automatic processes, two sets of tasks were identified: { A_SUBMITTED, A_PARTLYSUBMITTED } and { A_APPROVED, A_ACTIVATED, A_REGISTERED }. After extracting the causal relations and dependencies between events, we were able to confirm that in fact these tasks are redundant and can potentially contribute to an increase in operational costs and, consequently, to a decrease in productivity and efficiency. Considering Fig 3, we can see that after the root node *A*_*SUBMITTED*, the node *A*_*PARTLYSUBMITTED* always occurs. To extract a more efficient business process out of the data, we merged these two tasks into a single one and called it *A*_*START*_*APPLICATION*.

**Fig 4:**
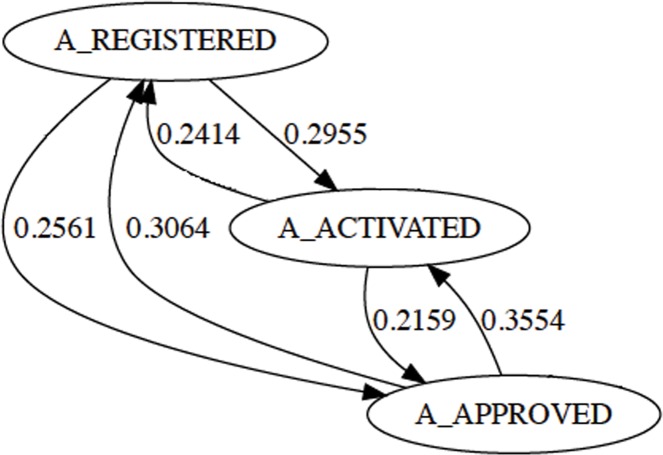
Redundancy found between events {A_APPROVED, A_ACTIVATED, A_REGISTERED}.

The same occurs for the ending processes (Fig 4). The dataset shows that before a credit application is approved, these three nodes occur interchangeably. Again, they are consuming extra and unnecessary resources and in order to reduce the complexity of the model, we merged these tasks into a single one: { A_APPROVED, A_ACTIVATED, A_REGISTERED } → A_CREDIT_APPROVED.

**Fig 5:**
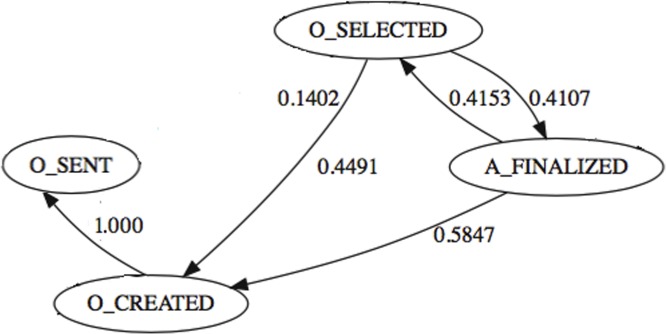
Redundancy found between events {O_SELECTED, O_CREATED, O_SENT}.

Finally, in Fig 5, the dataset shows that after an offer is created, the offer is always sent. Also, it seems that there are no rules in the application of the task *O*_*SELECTED*. Almost half of the times it is triggered by the finalization of the automatic process *A*_*FINALIZED*. At some other times, it is the task *O*_*SELECTED* that triggers the *A*_*FINALIZED* task. This last transition makes no real sense, because first the automatic processes are conducted and only then, if they are successful, the manual tasks and offer tasks start. Given this order inconsistency, it seems that this task has been subjected to human intervention. It is straightforward that an offer cannot be done before the application process is finalized, so we know that *A*_*FINALIZED* precedes the creation of the offer. To avoid redundancy and inconsistencies, we decided to group the three tasks into a single one called *O*_*OFFER*_*SENT*, that is { O_SELECTED, O_CREATED, O_SENT } → O_OFFER_SENT. We note that by removing these redundancies and unnecessary tasks, we were able to reduce the complexity of the business process from 24 to 18 tasks.

### 2.4 Elimination of cycles

The next step to optimise the business process is to eliminate cycles. This step plays an important role for two main reasons. First, it enables the discovery of cyclic sequences of tasks. Usually, these types of tasks are redundant and they contribute to the company’s inefficiency. This translates again into a decrease in productivity and a vast increase in operational costs and production (or service delivery) time. Second, the literature has reported the effectiveness of acyclic decision models as good approaches to model business processes and sequences of events [46]. A type of acyclic decision model that we are going to explore in this work are the Bayesian Networks [47].

**Fig 6:**
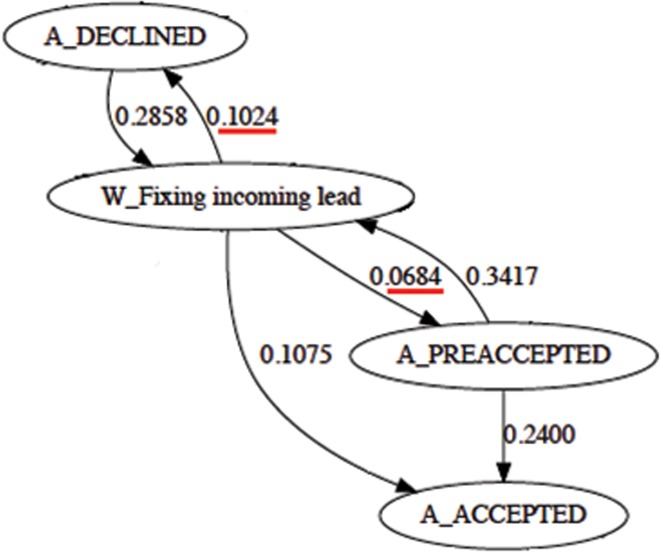
Subprocess containing a transition with a cycle. The transition with the lowest probability was removed in order to guarantee an acyclic structure.

These two reasons made us pursue the direction of eliminating cycles in the business process as a way to optimise the underlying processes that make up the bank. Fig 6, for instance, consists in a fragment of the business process, which contains cycles. One can easily notice that there could be human error between the transition of the manual task to the automatic task *W*_*Fixing*_*Incoming*_*Lead* → *A*_*PREACCEPTED* (which only contains a transition probability of 0.0684) versus the opposite direction *A*_*PREACCEPTED* → *W*_*Fixing Incoming Lead* (which has a probability of 0.3417). This actually makes some sense. Human worker’s tasks are more subject to human errors in contrast with pre-programmed computer automatized tasks. In these circumstances, we eliminate the cycle by simply deleting the edge with the lowest probability of occurrence. In Fig 6, the same reasoning can be made between tasks *W*_*Fixing*_*Incoming*_*Lead* and *A*_*DECLINED*.

### 2.5 Final network structure

In summarizing, to extract a network structure representing the underlying processes that make up the bank, we proceeded in the following way:

1. processing of the event log: identifying all tasks that were being conducted in the bank and determining the frequency of their occurrences. In the end, we identified 24 different tasks, contained in 262,200 events, which belonged to 13, 087 different loan applications
2. Extraction of a network structure, which initially was very complex to deal with due to the vast amount of transitions between tasks
3. Optimization of the network structure, which consisted of three main steps: (1) elimination of all edges with a transition probability below 0.05; (2) identification and elimination of redundant tasks and; (3) identification and elimination of cycles.

**Fig 7:**
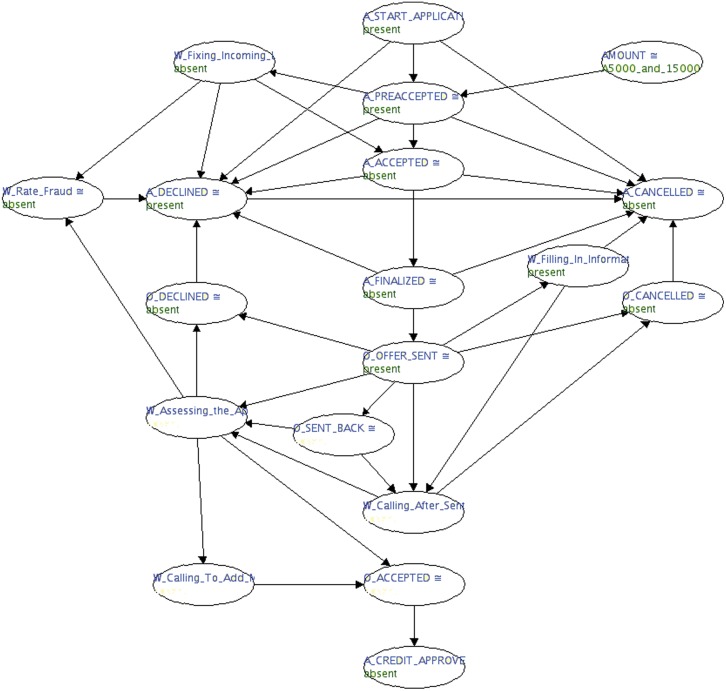
Optimised and reduced acyclic network structure extracted from the loan application bank event log.

In the end, we obtained a clear acyclic graphical structure (Fig 7) representative of the business processes that makes up the bank from the beginning of a loan application until its end (either with a successful outcome or a denial). This structure is clearer and can now be analysed in terms of probabilistic inferences.

Given the acyclic structure of the network, the next step is to fill the corresponding conditional probability table, which shows the probability distribution of a random variable given its parents nodes. In the next section, we briefly explain how this was achieved.

## 3 Learning the conditional probabilities

The acyclic network structure that we obtained from the event log is called a Bayesian Network. Bayesian networks are probabilistic graphical models that are used to model decision scenarios. They aid in making probabilistic inferences, that is, asking queries to the model and receiving answers in the form of probability values.

Under the realm of process mining, Bayesian Networks can represent activities as nodes (i.e. random variables) and the edges between activities can be seen as transitions between these tasks. From this structure, it is possible to automatically learn the conditional probability tables from a complete log of events using statistical models. Every node of the network is associated with a conditional probability table, which specifies the probability distribution of a node, given its parents nodes.

**Fig 8:**
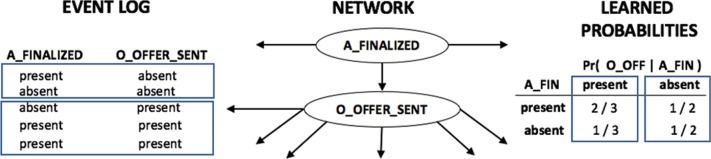
Example of learning a conditional probability table from a complete dataset and a known network structure. The learning process consists in simply counting the number of occurrences of each assignment of the random variables and normalizing the final counts to obtain a probability value.

Having a complete network structure, the estimation of the probabilities of a node given its parents nodes is straightforward. The financial institution provided a complete sample of their event log. When we have a known network structure and a full dataset, then the conditional probabilities of the network can be computed by simply counting how many times the conditioned variables occurred in the dataset. For instance, in the example in Fig 8, the variable *O*_*OFFER*_*SENT* has one single parent node, *A*_*FINALIZED*. Both variables are binary and can represent the *presence* or *absence* of the event: if the task *A*_*FINALIZED* has been executed, then it is *present*, otherwise it is *absent* from the application form.

Using the example in Fig 8, the learning process of a conditional probability table from a complete dataset with a known network structure simply consists in counting the number of occurrences of each assignment of the random variables and normalizing the final counts to obtain a probability value. When the variable *O*_*OFFER*_*SENT* has the value *present*, there are 2 out of 3 entries in the dataset where its parent variable also occurs (probability of 0.67) and 1 out of 3 entries where it does not (with probability 0.33). In the same way, when *O*_*OFFER*_*SENT* is *absent*, then we find that there is 1 out of 2 entries in the dataset where its parent variable is found to be *present* and *absent*, leading to a probability of 0.5.

One can see that the task of learning is very easy and straightforward in these circumstances. However, in most of the real world scenarios that is not the case. It is quite common that event logs are incomplete with several amounts of missing information (for instance, workers forget to register their tasks). The absence of data is translated into a drastic decrease of precision and compromises the statistical models, leading to biased and unrepresentative results.

For the study of this paper, which consists in comparing the effectiveness of quantum-like probabilistic inferences with classical inferences, it is straightforward to understand that for a complete dataset, the classical probabilistic inferences performed will always be more representative of the data, because we are learning the data in a classical way. The interesting question to explore is: what is the impact of quantum-like probabilistic inferences when the dataset is not robust enough and suffers from a vast amount of missing information (which is actually quite common in real world scenarios). In this situation, the classical statistical models cannot generalize well and will lead to inaccurate results.

**Fig 9:**
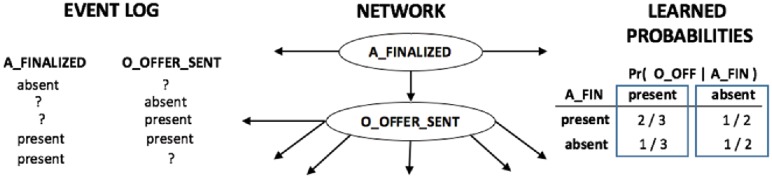
Example of learning a conditional probability table from an incomplete dataset and a known network structure. The learning process consists in the application of statistical methods that assume that events are distributed according to a Gaussian distribution (the Expectation/Maximisation algorithm).

To explore this condition, we randomly removed 70% of the data from the event log and used a learning algorithm called *Expectation / Maximization* to learn the conditional probability tables of the Bayesian Network [48]. Generally speaking, expectation / maximization is a statistical method. The mean and the variance of the probability distribution can be estimated by only knowing a partial sample of the dataset. The details of this algorithm already fall outside of the scope of this paper, but the reader can refer to the book of Bishop (2007) for further details. Fig 9, shows an example of what a dataset with missing data looks like and the final estimations of the conditional probability table learned with the expectation/maximization algorithm.

**Fig 10:**
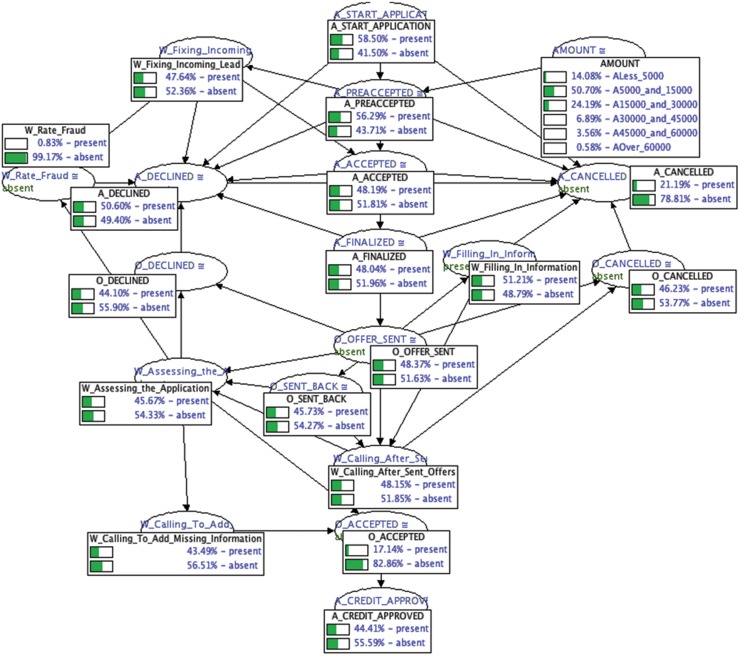
Resulting Bayesian network representing the business process of the financial institution with the conditional probability tables learned with 70% of the data missing.

**Fig 11:**
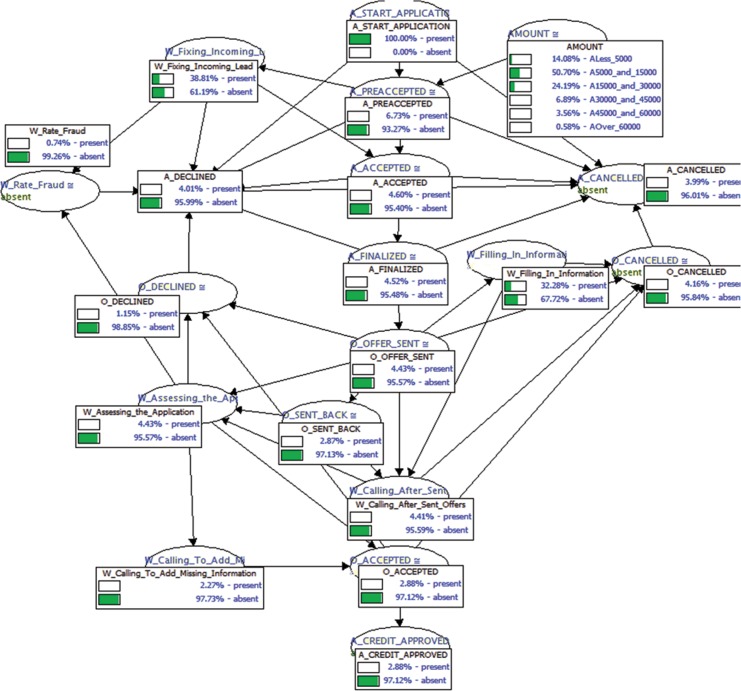
Resulting Bayesian network representing the business process of the financial institution with the conditional probability tables learned using the sull dataset.

It is interesting to notice that the conditional probabilities learned using the incomplete dataset do not reveal much information about the underlying business processes of the bank. The conditional probability tables learned for most of the tasks has nearly a 50% chance of either the task occurring or not. To give a more specific example, we can see that the probability of having a credit approved, *Pr*(*A*_*CREDIT*_*APPROVED*), is 44.41% in the Bayesian network learned with missing data (Fig 10). We contrast this with the 2.86% obtained in the Bayesian network with the conditional probability tables learned using the full dataset (Fig 11).

After finishing the learning phase, we ended up with two classical Bayesian networks: one for the missing data and another one for the full data represented in Figs 10 and 11, respectively. The Bayesian network in Fig 11 is our control network and will be used for evaluation purposes. Its conditional probability tables were learned using the full event log. On the other hand, the Bayesian network in Fig 10 is the one that will be used to compare classical inferences over quantum-like inferences and its conditional probability tables were learned using the same event log. However, 70% of its data was randomly missing, and therefore this introduced a high degree of uncertainty in the data.

**Fig 12:**
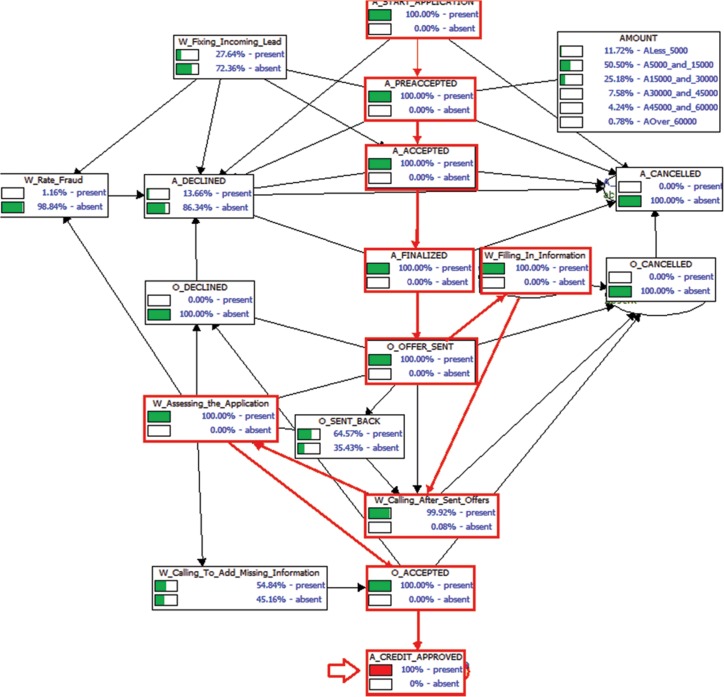
Impact of probabilistic inferences over Bayesian networks for process mining. Bayesian networks enables the specification of observed variables (evidence variables) and the specification of unobserved variables. In the figure, the only thing that was observed (piece of information provided) is that the variable *A*_*CREDIT*_*APPROVED* was observed to be *present*. With this piece of information, we can know the entire workflow of the company with 100% certainty.

At this stage one could be arguing about the effectiveness and applicability of Bayesian networks as appropriate decision models for process mining. Bayesian networks have already been used throughout the literature of business process management in many different scenarios [46]. In the literature, Markov chains are the most commonly used models to represent business processes [1]. However, Bayesian networks provide a different decision-making analysis in the sense that they enable the specification of evidence variables. In other words, they provide the specification of some knowledge about the decision scenario. For example, suppose that the only thing that we know about the state of the application process is that a credit was approved. Then, we can ask the network what the probability is of a certain task occurring (for instance, *W*_*Filling In Information*), given that we know that a credit was approved, *Pr*(*W*_*Filling In Information*|*A*_*CREDIT*_*APPROVED*). These types of inferences are unique to Bayesian networks and provide an interesting type of analysis that is not commonly performed in such type of decision scenarios. For instance, when we observe the state of the random variable *A_CREDIT_APPROVED = present*, then we know with certainty that the following events took place: *A_START_APPLICATION* → *A_PREACCEPTED* → *A_ACCEPTED* → *A_FINALIZED* → *O_OFFER_SENT* → *W_Filling_In_Information* → *W_Calling_After_Sent_Offers* → *W_Assessing_the_application* → *O_ACCEPTED* → *A_CREDIT_APPROVED* (Fig 12).

In the next section, we will formally present how to perform such types of probabilistic inferences both on classical and quantum-like Bayesian networks.

## 4 Exact inference in classical and quantum-like Bayesian network

Since the event logs of the bank are stored by their execution time, describing thus a causal sequence between events, we will explore the applicability and effectiveness of quantum-like Bayesian networks [33] in the prediction of several events from the loan application process. A quantum-like Bayesian network can be defined as an acyclic directed graph in which each node represents a random variable. Each edge represents a direct influence from the source node to the target node and uses probability amplitudes, which will be responsible for the emergence of interference effects. Moreover, Bayesian Networks allow us to deal with uncertainty: each task can either be present or absent in the business process. Therefore, it is possible to perform an analysis that will enable the computation of the probability of some task of the business process occurring, given that we do not know which tasks have already been performed [47].

### 4.1 Classical Bayesian networks

**Fig 13:**
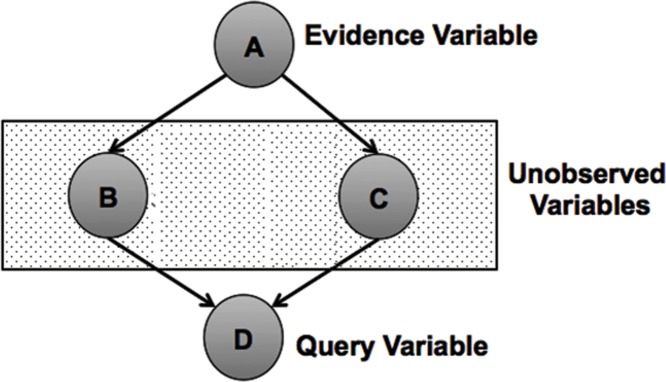
General example of a classical Bayesian network. Each node represent a random variable and each edge represents a direct influence from a source node to a target node. Each node is followed by a conditional probability table, which specifies the probaility distribution of a node given its parents.

A classical Bayesian network can be defined by a directed acyclic graph structure in which each node represents a different random variable from a specific domain and each edge represents a direct influence from the source node to the target node. The graph represents independence relationships between variables and each node is associated with a conditional probability table which specifies a distribution over the values of a node given each possible joint assignment of values of its parents. This idea of a node depending directly upon its parent nodes forms the core of Bayesian networks. Once the values of the parents are known, no information relating directly or indirectly to its parents or other ancestors can influence the beliefs about it [6]. Fig 13 shows an example of a classical Bayesian network.

#### 4.1.1 Classical full joint distributions

In classical probability theory, the full joint distribution over a set of *N* random variables *Pr*(*X*_1_, *X*_2_, …, *X*_*N*_) corresponds to the probability distribution assigned to all of these random variables occurring together in the same sample space [6]. The full joint distribution of a Bayesian network, where *X*_*i*_ is the list of random variables and *Parents*(*X*_*i*_) corresponds to all parent nodes of *X*_*i*_, is given by Eq 4 [49]

$$\begin{array}{c} Pr\left(X_{1},\ldots ,X_{n}\right)=\prod_{i=1}^{n}Pr\left(X_{i}|Parents\left(X_{i}\right)\right) \end{array}$$ (4)

#### 4.1.2 Classical marginalization

Given a query random variable *X* and let *Y* be the unobserved variables in the network, the marginal distribution of *X* is simply the probability distribution of *X* averaging over the information about *Y*. The marginal probability for discrete random variables, can be defined by Eq 5. The summation is over all possible *y*, i.e., all possible combinations of values of the unobserved values *y* of variable *Y*. The term *α* corresponds to a normalization factor for the distribution *Pr*(*X*) [49].

$$\begin{array}{c} \begin{array}{c} Pr\left(X=x\right)=\alpha \sum_{y}Pr\left(X=x|Y=y\right)Pr\left(Y=y\right)\text{,}\;\;\;\;\;\;\;\;\;\;\text{where}\;\alpha =\frac{1}{\sum_{x\in X}Pr\left(X=x\right)} \end{array} \end{array}$$ (5)

### 4.2 Quantum-like Bayesian networks

**Fig 14:**
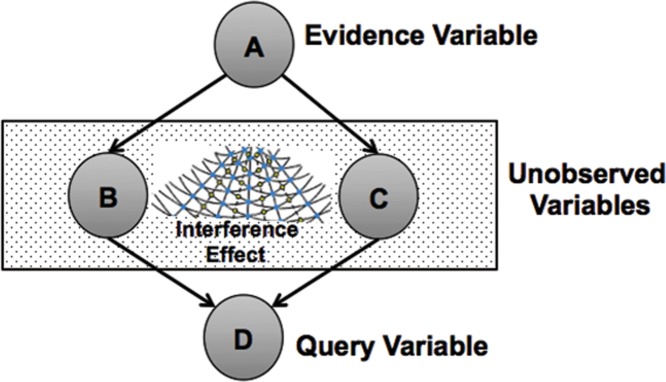
General example of a quantum-like Bayesian network. Each node represent a random variable and each edge represents a direct influence from a source node to a target node. Unobserved nodes can produce quantum interference effects, which can disturb the final probability outcomes.

A quantum-like Bayesian Network can be defined by a directed acyclic graph structure in which each node represents a different random variable and each edge represents a direct influence from the source node to the target node. The graph can represent independence relationships between variables, and each node is associated with a conditional probability table that specifies a distribution of probability amplitudes over the values of a node given each possible joint assignment of values of its parents. In other words, a quantum-like Bayesian Network is defined in the same way as a classical network with the difference that probability values are replaced by probability amplitudes (as we remarked before those amplitudes are complex valued) [33]. Fig 14 shows an example of a classical Bayesian network.

#### 4.2.1 Quantum-like full joint distribution

The quantum-like full joint probability distribution can be defined in the same way as in a classical setting with two main differences: (1) the probability values are replaced by probability amplitudes and; (2) the probability value is given by applying the squared magnitude of a projection. In this sense, the quantum-like full joint probability amplitude distribution over a set of *N* random variables *ψ*(*X*_1_, *X*_2_, …, *X*_*N*_) corresponds to the probability distribution assigned to all of these random variables occurring together in a Hilbert space. Then, the full joint probability amplitude distribution of a quantum-like Bayesian Network is given by

$$\begin{array}{c} \psi \left(X_{1},\ldots ,X_{N}\right)=\prod_{j=1}^{N}\psi \left(X_{j}|Parents\left(X_{j}\right)\right) \end{array}$$ (6)

Note that, in Eq 6, *X*_*i*_ is the list of random variables (or nodes of the network), *Parents*(*X*_*i*_) corresponds to all parent nodes of *X*_*i*_ and *ψ*(*X*_*i*_) is the probability amplitude associated with the random variable *X*_*i*_. The probability value is extracted by applying Born’s rule, that is, by making the squared magnitude of the joint probability amplitude, *ψ*(*X*_1_,…, *X*_*N*_)

$$\begin{array}{c} Pr\left(X_{1},\ldots ,X_{N}\right)={\left|\psi (X_{1},\ldots ,X_{N})\right|}^{2} \end{array}$$ (7)

#### 4.2.2 Quantum-like marginalization

The quantum-like marginalization formula is the same as the classical one with two main differences: (1) the probability values are replaced by probability amplitudes; (2) the probability is obtained by applying Born’s rule. More formally, given a query random variable *X* and let *Y* be the unobserved variables in the network, the marginal distribution of *X* is simply the probability amplitude distribution of *X* averaging over the information about *Y*. The quantum-like marginal probability for discrete random variables can be defined by Eq 8. The summation is over all possible *y*, i.e. all possible combinations of values of the unobserved values *y* of variable *Y*. The term *γ* corresponds to a normalization factor. Since the conditional probability tables used in Bayesian networks are not unitary operators with the constraint of double stochasticity (like it is required in other works of the literature [50, 51]), we need to normalize the final scores. In classical Bayesian inference, on the other hand, normalization is performed due to the independence assumption made in Bayes’ rule

$$\begin{array}{c} Pr\left(X|e\right)=\gamma {\left|\sum_{y}\prod_{k=1}^{N}\psi \left(X_{k}|Parents\left(X_{k}\right),e,y\right)\right|}^{2} \end{array}$$ (8)

Note that double stochasticity of a square matrix requires that each row and each column of non-negative real numbers adds up to one. Expanding Eq 8 will lead to the quantum-like marginalization formula [52], which is composed of two parts: one representing the classical probability and the other representing the interference term (which corresponds to the emergence of destructive / constructive interference effects)

$$\begin{array}{c} Pr\left(X|e\right)=\gamma \sum_{i=1}^{\left|Y\right|}{\left|\prod_{k}^{N}\psi \left(X_{k}|Parents\left(X_{k}\right),e,y=i\right)\right|}^{2}+2\cdot Interference \end{array}$$ (9)

$$\begin{array}{c} Interference=\\ \sum_{i=1}^{|Y|-1}\sum_{j=i+1}^{\left|Y\right|}|\prod_{k}^{N}\psi \left(X_{k}|Parents\left(X_{k}\right),e,y=i\right)|\cdot |\prod_{k}^{N}\psi \left(X_{k}|Parents\left(X_{k}\right),e,y=j\right)|\cdot \cos\left(\theta_{i}-\theta_{j}\right) \end{array}$$

Note that, in Eq 9, if one sets (*θ*_*i*_ − *θ*_*j*_) to *π*/2, then cos(*θ*_*i*_ − *θ*_*j*_) = 0. This means that the interference term is canceled and the quantum-like Bayesian network collapses to its classical counterpart. In other words, one can see the quantum-like Bayesian Network as a more general and abstract model of the classical network, since it represents both classical and quantum-like behaviour. Setting the angles to right angles means that all cosine similarities are either 0 or 1, transforming a continuous-valued system to a Boolean-valued system. Moreover, if the Bayesian network has *N* binary random variables, we will end up with 2^*N*^ free *θ* parameters, which is the size of the full joint probability distribution.

It remains an open question to come up with a formal method to assign values to interference terms. However, some work has already been done in that direction [33, 34, 53]. In this paper, we will use the heuristic developed in the work of [33] in order to set the interference parameters.

### 4.3 Interference terms

So far, we presented a general quantum-like Bayesian network model, which performs quantum-like probabilistic inferences. In the recent work of [33], the authors propose a similarity heuristic, which proves to be effective in paradoxical scenarios that are violating the Sure Thing Principle [54]. The Sure Thing principle plays a key role in the Ellsberg paradox we mentioned in section 1 of this paper. Note that a heuristic is simply a shortcut that generally provides good results in many situations (in this case, for violations to the Sure Thing Principle), but at the cost of occasionally not giving us very accurate results [55].

**Fig 15:**
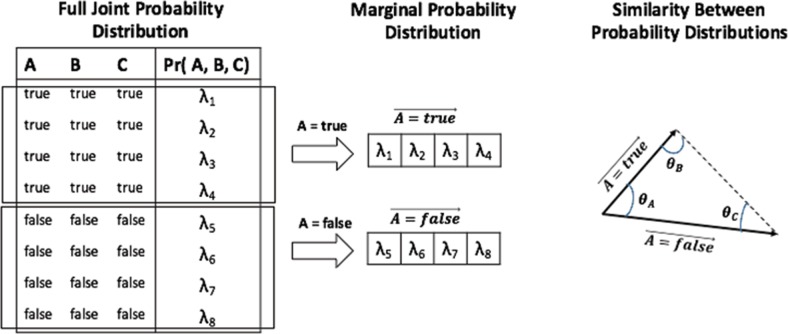
Example of how to compute the similarity heuristic proposed in the previous work of [33].

Probabilistic inferences are computed by selecting from the full joint probability distribution the appropriate assignments. Following the example in Fig 15, if we want to compute the probability of the random variable *A* being *true*, *Pr*(*A* = *true*), then one selects from the full joint probability distribution all the entries where *A* = *true* and all entries where *A* = *false*. These entries correspond to the marginal probability distribution, and if we sum the values of the vectors and normalize them, we will end up with a classical probability to the query

$$\begin{array}{c} Pr\left(A=true\right)=\alpha \sum_{i=1}^{N}\lambda_{i}, \end{array}$$

where *α* is the normalization factor. If we add an interference term to this formula, then we will end up with a quantum-like probability answer to the same query, *γ* being the normalization factor

$$\begin{array}{c} Pr\left(A=true\right)=\gamma (\sum_{i=1}^{N}\lambda_{i}+2\sum_{i=1}^{N-1}\sum_{j=i+1}^{N}\sqrt{\lambda_{i}}\sqrt{\lambda_{j}}Cos(\theta_{i}-\theta_{j})) \end{array}$$

The interference parameters *θ* are obtained by extracting the similarity values between the marginal distribution vectors. This is achieved by computing the cosine similarity between them, which is a widely used similarity function in information retrieval [56]. Following Fig 15, the cosine similarity will gives us three degrees of similarity between the vectors: *θ*_*A*_, *θ*_*B*_ and *θ*_*C*_. In the work of [33], the authors created the similarity measure *ϕ*, which is given by the ratio between the angles of the probability vectors

$$\begin{array}{c} \phi =\frac{\left(\theta_{C}-\theta_{B}\right)}{\theta_{A}} \end{array}$$

Note that *ϕ* is obtained based on the marginal probability distribution of the data. It measures the relation between two probability values, because we are considering binary random variables, and nothing else.

Just like ‘learning’ algorithms need to *learn* the distribution of the data, in the quantum-like Bayesian network we also need to perform an analysis of the data in order to set the interference terms. Usually, one needs to have prior knowledge of the outcome of a decision scenario and only then can one manually adjust the interference effects [31, 50, 51]. This is feasible for very small and controlled decision scenarios, however when we move to large scale and complex decision scenarios with millions of parameters to set, this approach is intractable.

The similarity heuristic proposed by [33] requires the definition of some threshold values based on the similarity measure *ϕ*. In their work, the authors were able to obtain proper thresholds to predict many different experiments, which were violating the Sure Thing Principle. Since we are not dealing with violations to the Sure Thing Principle in this paper, we performed a preliminary analysis of the data in order to establish and learn the thresholds (or boundaries) of the heuristic function. The function devised is represented in Eq 10.

$$\begin{array}{c} h_{\theta }=\{\begin{array}{cc} 1.5408\;\;\;\;\;\;\;\text{if}\;\phi <-2 & \\ 1.5178\;\;\;\;\;\;\;\text{if}\;\phi >=-2\;\;\&\&\;\;\phi <=0 & \\ \pi \;\;\;\;\;\;\;\;\;\;\;\;\;\;\;\;\text{if}\;\phi >=0.15 & \\ 0\;\;\;\;\;\;\;\;\;\;\;\;\;\;\;\;\text{otherwise} & \end{array} \end{array}$$ (10)

It is important to note that both classical and quantum-like models have the same amount of information: they only use the marginal probability distribution. The difference relies in the fact that classical probability uses real numbers and quantum-like models use complex numbers, which will lead to the emergence of the interference effects that can be anything in a given range of values. This is also a reason why we need to specify these thresholds in the heuristic function, otherwise we would have no control over the interference terms. Appendix B presents in more detail how to compute the similarity heuristic for quantum-like inferences.

## 5 Comparison between classical and quantum-like Bayesian networks

After learning the conditional probabilities of the Bayesian network and after presenting the inference process in Bayesian networks (both classical and quantum-like), we will now proceed with a comparison of the probabilistic inferences obtained in both classical and quantum-like Bayesian networks in the scenario where 70% of the data from the event log is missing.

We want to emphasize the point that randomly removing 70% of the data is akin to simulating a real world situation. Although the full dataset was kindly provided by a Dutch bank, we have argued in this paper that in real world scenarios, financial data suffers from the problem of incomplete data [57]. This also provides for a rationale why there is an increasing need to use machine learning algorithms to generalize information based on a sample of data [58].

In order to compare classical probabilistic inferences with quantum-like inferences in the Bayesian network with missing data, we queried each variable of the Bayesian network and compared the outcome with a Bayesian network whose conditional probability tables were learned using the full data of the event log.

The results of comparing the probabilistic inferences performed in a Bayesian network with classical and quantum-like inferences are detailed in Table 4.

**Table 4:** Comparison between quantum-like and classical inferences over a Bayesian network learned using an incomplete dataset (with 70% of missing data). The results show that quantum-like inferences achieved an average error of 5.90% when compared to the 22.85% error obtained in the classical inference. The column COMPLETE DATA BN represents the control network, which was learned using the full dataset.

	MISSING DATA BN				COMPLETE DATA BN
	Inferences		Error (%)		Inferences
	Quantum	Classical	Quantum	Classical	Classical (baseline)
**Pr(A_PREACCEPTED = present)**	**0.0787**	0.3298	**1.14**	26.25	0.0673
**Pr(A_ACCEPTED = present)**	**0.0292**	0.3152	**1.75**	26.85	0.0467
**Pr(A_CREDIT_APPROVED = present)**	**0.0110**	0.1674	**2.01**	13.86	0.0311
**Pr(A_DECLINED = present)**	**0.5325**	**0.5325**	**3.88**	**3.88**	0.5713
**Pr(A_FINALIZED = present)**	**0.0286**	0.1786	**1.73**	13.28	0.0458
**Pr(O_SENT_BACK = present)**	**0.1022**	0.4115	**7.31**	38.24	0.0291
**Pr(O_CANCELLED = present)**	**0.1760**	0.4160	**17.23**	41.23	0.0037
**Pr(O_DECLINED = present)**	**0.0431**	0.4070	**3.81**	40.20	0.0050
**Pr(O_ACCEPTED = present)**	**0.0110**	0.1674	**2.01**	13.86	0.0311
**Pr(O_OFFER_SENT = present)**	**0.0588**	0.1014	**1.39**	5.71	0.0449
**Pr(W_Assessing_the_Application = present)**	**0.0643**	0.4160	**1.98**	37.15	0.0445
**Pr(W_Calling_After_Sent_Offers = present)**	**0.0405**	0.4177	**0.44**	37.28	0.0449
**Pr(W_Calling_To_Add_Missing_Info = present)**	**0.0305**	0.4297	**0.77**	40.69	0.0228
**Pr(W_Fixing_Incoming_Lead = present)**	**0.4006**	0.4792	**1.25**	9.11	0.3881
**Pr(W_RATE_FRAUD = present)**	0.0019	**0.0082**	0.55	**0.08**	0.0074
**Pr(W_Filling_In_Information = present)**	0.5372	**0.4706**	21.40	**14.74**	0.3232
**Pr(A_CANCELLED = present)**	0.0749	**0.1260**	31.63	**26.52**	0.3912

The results show that the quantum-like inferences were able to adjust the probabilistic inferences of the classical network in scenarios with high levels of uncertainty (no variables observed). One can interpret quantum-like probabilistic inferences as an additional layer to the classical inferences that allows for a non-linear parameterisation of the data.

It is interesting to note that quantum-like inferences either outperform classical inferences or, in a worst case scenario, have the same performance as a classical network. This issue has already been noticed and pointed out in the previous studies of [33, 52, 59]. The queries performed over the random variables *A*_*FINALIZED*, *A*_*CANCELLED* and *W*_*FixingIncomingLead* were the ones with higher errors, but they had nearly the same performance as the classical network. The quantum-like model achieved a mean error of 17.86% compared with the 13.78% mean error obtained in a classical setting. The general results show that the average error over the 19 random variables was, in scenarios where nothing is observed, for the quantum-like Bayesian network 5.90% compared to a 22.85% error in the classical network.

A statistical analysis was also performed where we used a paired t-test to attest the significance of the results. Table 5, shows that the probabilistic inferences obtained in the quantum-like Bayesian Network were statistically significant for a confidence level of 95%.

**Table 5:** Paired sample t-test of the inference computed by the classical BN compared to the inferences computed by the quantum-like BN. Results show that the average inferences computed by the quantum-like BN were statistically significant for a confidence interval of 95%.

Mean	St Deviation	St Error Mean	Conf. Inter. 95%	t-value	p-value	Significant?
0.1695	15.7620	3.8228	[8.845; 25.0530]	4.434	0.0004 < 0.05	**yes**

Although much more research needs to be done, this study suggests that quantum-like inferences could be used as a way to complement inferences in classical models. This can have high impact in several domains where machine learning plays an important role (for instance, in medical decision-making or even in portfolio optimisation [60]).

### 5.1 Advantages and disadvantages of quantum-like Bayesian networks

It is straightforward that quantum-like Bayesian networks suffer the same problem of the exponential increase of complexity (expressed as the dimension of the state space) as the classical Bayesian networks. Indeed, in what concerns the complexity of the inference problem, Bayesian networks (either classical or quantum-like) will always be *NP-Hard*. This means that exact inference on Bayesian networks are part of a class of problems that are extremely hard for a computer to solve, because it takes an exponential number of computational steps to perform the computations. The hardness of the exact inference comes precisely in the computation of the full joint probability distribution, which takes at most 2^*N*^ − 1 (*N* being the number of nodes in the network) computational steps assuming that all random variables of the network are binary. This gives a complexity of *O*(2^*N*^). If random variables are not binary, then the exact inference process becomes even worse with a complexity of *O*(*M*^*N*^), where *M* is the number of assignments that the random variables can have.

The initial analysis that we performed in this paper enabled us to identify redundant tasks in a bank. With the help of a preliminary analysis, we were able to decrease the number of tasks in the business process from 25 events to 19. In order to gain some idea of the impact of this identification in the inference problem, we can say the following. If we used all tasks that were identified in the event log, we would end up with a full joint probability distribution of 6 × 2^23^ = 50, 331, 648 entries, which corresponds to the *AMOUNT* random variable (which contains 6 different assignments) and 23 binary random variables (which contains 2^23^ different assignments). Under a classical setting, this is computationally intractable and in order to deal with this situation we could not use exact inference mechanisms. An alternative approach would be the use of approximative inference methods, such as the belief propagation algorithm originally proposed by [47]. However, quantum-like versions of this algorithm have not been heavily explored in the literature. With the identification of the redundant tasks, we were able to reduce the state space to 6 × 2^19^ = 3, 145, 728 entries, which is already computationally tractable.

The quantum-like Bayesian network suffers from the same problem as the classical network in terms of the exponential increase of the full joint probability distribution. However, it also enables a new set of free parameters, which are the consequence of the interference effects. These interference effects can be seen as an additional non-linear parametrical layer that is added to classical inferences in order to refine probabilistic inferences. A preliminary analysis of the data needs to be performed in order to refine the boundaries that are required for the heuristic proposed in [33]. The computation of these quantum interference effects can be performed in quadratic time with an addition of *m*(*m* + 1)/2*m* operations, where *m* is the size of the marginal probability distribution. In the end, we lose a little bit of performance, but we are able to get a decision model which relative to the classical network, provides for a better representation of a decision scenario under high levels of uncertainty.

All simulations, the Bayesian networks and the code to perform classical and quantum-like inferences that we used in the experimental findings of this work will be made freely available for researchers (https://github.com/catarina-moreira/bpmn).

## 6 Conclusions

In this paper, we investigated how classical probabilistic models are affected by incomplete event logs and we explored quantum-like probabilistic inferences as an alternative mathematical model to classical probability. We presented a pioneering study which studies the impact of interference terms in a real life, large scale decision scenario. This work also showed that Bayesian Networks provide an interesting analysis of a business process, since it represents the uncertainty differently from the traditional models of the literature (like Markov Chains or Petri Nets). While in Markov Chains business processes are modelled as a chain of events that are observed to occur, under a Bayesian Network perspective, this does not apply: each task can either be present or absent in the business process. Therefore, Bayesian Networks allows the modelling of uncertainty associated with a business process by performing a different analysis that will enable the computation of the probability of some task of the business process occurring, given that we do not know which tasks have already been performed.

We analysed a loan applications dataset from a Dutch bank. We were able to discover the underlying processes that make up the bank’s business processes and we optimised the workflow by identifying redundant tasks and insignificant sequences of tasks. Data is usually missing or unreliable and, in the absence of data, statistical methods cannot come up with a general model representative of the data. For this reason, it is important to employ novel methods that are capable of dealing with incomplete datasets and uncertainty.

Quantum-like models have proven throughout the literature that they are capable of representing uncertainty in a more general way than classical models, due to the usage of quantum interference effects. These interference effects can be seen as an additional non-linear parametrical layer that is added to classical inferences in order to refine probabilistic inferences. The drawback is that a preliminary analysis of the data needs to be performed in order to refine the boundaries that are required for the similarity parameter in the heuristic we discussed. Also, the computation of these quantum interference effects can be performed in quadratic time. We lose a little bit of performance, but we gain in terms of accuracy. So far, quantum-like models have only been applied in very small and controlled experiments. The study conducted in this paper represents a first attempt to assess the effectiveness of quantum-like models in real life scenarios. From this work, we verified that under large and complex decision scenarios with high levels of uncertainty, quantum-like inferences were able to outperform classical inferences.

## Appendix

## A Inferences in quantum-like Bayesian networks

The quantum-like Bayesian Network proposed in [33] is built in a similar way as a classical network, with the difference that it uses complex amplitudes to specify the conditional probability tables, instead of real probability values. As a consequence, the quantum-like Bayesian Network will give rise to interference effects, which can act destructively or constructively if the interferences are negative or positive, respectively.

Algorithm 1 describes the main steps to compute quantum-like inferences. Basically, a probabilistic inference consists of two major steps: the computation of the full joint probability distribution of the network and the computation of the marginal probability distribution with respect to the variable being queried.

The algorithm starts by receiving a Bayesian network represented by a set of *factors* specified by probability amplitudes instead of probability values. A factor is a function that takes as input a set of random variables and returns all the assignments corresponding to that random variable. For instance, the full joint probability distribution of a network can be seen as a factor. The algorithm also receives a set of observed variables if some conditional probability is being queried. The random variable to be queried is also received as input.

Given a Bayesian network represented as a set of factors, the algorithm first checks if there are any observed variables. More specifically, if the probabilistic inference is conditioned on some observed variable(s), then, for computational reasons, we set the values of the conditional probability tables, which are *not* consistent with the observed variables to 0. By doing so, we are computing just the probabilities of the joint probability distribution that matter for the inference process, instead of computing the entire full joint probability distribution table.

Next, we compute the full joint probability distribution. This corresponds to the application of the full joint probability distribution formula described in Eq 6. Basically, this function performs the product for each assignment of all random variables of the network. One needs to guarantee that the full joint probability distribution obeys the normalization axiom, making all entries of the distribution sum to one.

Having the full joint distribution factor, we can perform the probabilistic inference by computing the classical marginal probability distribution and the interference term. The function *FactorMarginalization* corresponds to the selection of all entries of the full joint probability distribution that match the query variable and the evidence variables (if given). It returns two vectors: (1) one corresponding to the entries of the full joint probability where the query variable is observed to occur (we address these probabilities as *PositiveProb*); and (2) another one corresponding to the entries of the full joint probability where the query variable is observed to not occur (*NegativeProb*). The classical probability corresponds to a normalized summation of these vectors.

Having the vectors with the positive and negative probabilities resulting from the marginalization process, we can also compute the quantum-like probabilities (Algorithm 2). The quantum interference formula in Eq 9 is given by the set of two summations over the marginal probability vector. Due to normalization purposes, we will need to compute the quantum interference term corresponding both to the positive and negative probability measures (when the query variable occurs and not occurs). The quantum interference parameter *θ* is computed according to the similarity heuristic and will be addressed with more detail in Section B of this Appendix.

## B The similarity heuristic for interference effects

The goal of the similarity heuristic is to determine an angle between the probabilistic vectors associated with the marginalization of the positive and negative assignments of the query variable. In other words, when performing a probabilistic inference from a full joint probability distribution table, we select from this table all probabilities that match the assignments of the query variable. If we sum these probabilities, we end up with a final classical probability inference. If we add an interference term to this classical inference, we will end up with a quantum-like inference. In this case, we can use these probability vectors to obtain additional information to compute the interference parameters. The general idea of the similarity heuristic is to use the marginal probability distributions as probability vectors and measure their similarity through the law of cosines formula, which is a similarity measure well known in the Computer Science domain and widely used in Information Retrieval [56]. According to this degree of similarity, we will apply a mapping function with a heuristic nature, which will output the value for the interference parameter *θ* by taking into consideration a previous study of the probabilistic distribution of the data of several experiments as reported in the literature.

**Algorithm 1** Quantum-Like Bayesian Network

**Require**: F, factor structure

    *ObservedVars*, list of observed variables,

    *QueryVar*, identifier of the variable to be queried,

**Ensure**: Factor *Q*, corresponding to the quantum inferences,

    Factor *C*, corresponding to the classical inferences

1: /* A factor is a structure containing three lists:

  *var*, corresponds to an identifier of a random variable. It also contains the list of the parent vars.

  *card*, corresponds to the cardinality of each random variable in var.

  *val*, corresponds to the respective conditional probability table. */

2: *Q* ← *struct*(′*var*′, *QueryVar*,′ *card*′, 2, *val*, {});       // initialise output factor

 structure for quantum network

3: *C* ← *struct*(*var*, *QueryVar*,′ *card*′, 2, *val*, {});       // initialise output factor

 structure for classical network

4: // Observe evidence: set to 0 all factors in F that do not correspond to the evidence variables

5: F ← *ObserveEvidence*(F, *ObservedVars*);

6: // Compute the Full Joint Probability Distribution of the Network:

7:

$$\psi \left(X_{1},\ldots ,X_{N}\right)=\prod_{j=1}^{N}\psi \left(X_{j}|Parents\left(X_{j}\right)\right)$$

8: *Joint* ← *ComputeFullJointDistribution* (F);

9: // Marginalise the full joint probability distribution. Select the positive and negative assignments of *QueryVar*:

10: [*PositiveProb*, *NegativeProb*] ← *FactorMarginalization*(*Joint*, *QueryVar*);

11: // Compute classical probability factor by applying Eq 5

12: *C*.*val* ← *ComputeClassicalProb*(*PositiveProb*, *NegativeProb*);

13: // Compute quantum probability factor according to Algorithm 2

14: *Q*.*val* ← *ComputeQuantumProb*(*PositiveProb*, *NegativeProb*);

15: **return** [*Q*, *C*];

When performing quantum-like probabilistic inferences, two steps are required: (1) the computation of a quantum-like full joint probability distribution and; (2) the computation of the quantum-like marginal distribution. The superposition vector, comprising all possible events, is given by the full joint probability distribution already presented in Eq 6.

Algorithm 3 presents the pseudo-code of the proposed heuristic. Given two vectors: (1) one corresponding to the entries of the full joint probability where the query variable is observed to occur (we address these probabilities as *PositiveProb*) and; (2) another one corresponding to the entries of the full joint probability where the query variable is observed to not occur (*NegativeProb*). Then, one can compute the similarity heuristic in the following way.

**Algorithm 2** ComputeQuantumProbability

**Require**: *PositiveProb*, vector of marginal probabilities when *QueryVar* occurs,

    *NegativeProb*, vector of marginal probabilities when *QueryVar* does not occur,

**Ensure**: List *Q* with probabilistic inference using quantum theory

1: *interference*_*pos* ← 0;

2: *length*_*assign* ← *length*(*PositiveProb*);

3: // For all probability assignments,

4: **for** *i* = 1; 1*i* ≤ *length*_*assign* − 1; *i* = *i* + 1 **do**

5:  **for** *j* = *i* + 1; *j* ≤ *length*_*assign*; *j* = *j* + 1 **do**

6:   // Compute the quantum interference parameter *θ* according to a given heuristic function

7:   *heurs* ← *SimilarityHeuristic*(*PosAssign*, *NegAssign*)

8:   // Apply quantum interference formula:

9:  

$$\sum_{i=1}^{|Y|-1}\sum_{j=i+1}^{\left|Y\right|}|\prod_{k}^{N}\psi \left(X_{k}|Parents\left(X_{k}\right),e,y=i\right)|\cdot$$

        

$$\cdot |\prod_{k}^{N}\psi \left(X_{k}|Parents\left(X_{k}\right),e,y=j\right)|\cdot \cos\left(\theta_{i}-\theta_{j}\right);$$

10:   // Compute the interference term related to the positive assignments

11:   *interference*_*pos* ← *interference*_*pos* + 2*PosAssign* [*i*] *PosAssign* [*j*] *heurs*

12:

13:   // Compute the interference term related to the negative assignments (for normalisation)

14:   *interference*_*neg* ← *interference*_*neg* + 2*NegAssign* [*i*] *NegAssign* [*j*] *heurs*

15:  **end for**

16: **end for**

17: // Compute quantum-like probabilities: *classicalProb* + *interference*.

18: *α* = (*sum*(*PosAssign*) + *sum*(*NegAssign*))^−1^

19: *classicalProb* ← [*α PosAssign*, *α NegAssign*];

20: *probPos* ← *classicalProb*[1] + *interference*_*pos*;

21: *probNeg* ← *classicalProb*[2] + *interference*_*neg*;

22: // Normalise the results in order to obtain a probability value

23: *γ* ← (*probPos* + *probNeg*)^−1^

24: *Q* ← [*γ probPos*, *γ probNeg*]

25: **return** *Q*;

First, one computes the euclidean distances between both vectors. Having the distances, one can use the law of cosines measure to determine the angles between all these vectors. With all this information, one can compute the similarity measure *φ* of the vectors and get the output of the interference parameter. In the end, the algorithm returns the cosine of this value.

**Algorithm 3** SimilarityHeuristic

**Require**: *PositiveProb*, vector of marginal probabilities when *QueryVar* occurs,

    *NegativeProb*, vector of marginal probabilities when *QueryVar* does not occur,

**Ensure**: *inter f*, Quantum Interference term

1: // Compute Euclidean distances between vectors

2: *norm*_*c*_ ← *norm*(*PosProb* − *NegProb*, 2);

3: *norm*_*a*_ ← *norm*(*PosProb*, 2);

4: *norm*_*b*_ ← *norm*(*PosNeg*, 2);

5: // Compute angles between vectors using the law of cosines

6: $\theta_{a}\leftarrow ACos\left(\frac{norm_{b}^{2}-norm_{a}^{2}+norm_{c}^{2}}{2\;norm_{c}\;norm_{b}}\right);$

7: $\theta_{b}\leftarrow ACos\left(\frac{norm_{a}^{2}-norm_{b}^{2}+norm_{c}^{2}}{2*norm_{c}*norm_{a}}\right)$

8: $\theta_{c}\leftarrow ACos\left(\frac{norm_{a}^{2}+norm_{b}^{2}-norm_{c}^{2}}{2*norm_{a}*norm_{b}}\right);$

9: // Compute de similarity measure *φ*

10: $\phi \leftarrow \frac{\theta_{c}}{\theta_{a}}-\frac{\theta_{b}}{\theta_{a}};$

11: // Apply heuristic using the thresholds according to Eq 10

12: *inter f* ← 0;

13: **if** *ϕ* < −2 **then**

14:  *inter f* ← 1.5408;

15: **end if**

16: **if** *ϕ* > = −2 && *ϕ* < = 0 **then**

17:  *inter f* ← 1.5178

18: **end if**

19: **if** *ϕ* > = 0.15 **then**

20:  *inter f* ← *π*

21: **end if**

22: **return** *Cos*(*inter f*);
